# The Conserved Cys-2232 in *Clostridioides difficile* Toxin B Modulates Receptor Binding

**DOI:** 10.3389/fmicb.2018.02314

**Published:** 2018-10-26

**Authors:** Soo-Young Chung, Dennis Schöttelndreier, Helma Tatge, Viola Fühner, Michael Hust, Lara-Antonia Beer, Ralf Gerhard

**Affiliations:** ^1^Institute of Toxicology, Hannover Medical School, Hanover, Germany; ^2^Department of Biotechnology, Institute for Biochemistry, Biotechnology and Bioinformatics, Technische Universität Braunschweig, Braunschweig, Germany

**Keywords:** *Clostridioides difficile*, toxins, receptor binding, autoproteolysis, neutralization, antibody phage display

## Abstract

*Clostridioides difficile* toxins TcdA and TcdB are large clostridial glucosyltransferases which are the main pathogenicity factors in *C. difficile*-associated diseases. Four highly conserved cysteines are present in all large clostridial glucosyltransferases. In this study we focused on the conserved cysteine 2232 within the combined repetitive oligopeptide domain of TcdB from reference strain VPI10463 (clade I). Cysteine 2232 is not present in TcdB from hypervirulent strain R20291 (clade II), where a tyrosine is found instead. Replacement of cysteine 2232 by tyrosine in TcdB_V PI10463_ reduced binding to the soluble fragments of the two known TcdB receptors, frizzled-2 (FZD2) and poliovirus receptor-like protein-3/nectin-3 (PVRL3). In line with this, TcdB_R20291_ showed weak binding to PVRL3 in pull-down assays which was increased when tyrosine 2232 was exchanged for cysteine. Surprisingly, we did not observe binding of TcdB_R20291_ to FZD2, indicating that this receptor is less important for this toxinotype. Competition assay with the receptor binding fragments (aa 1101–1836) of TcdB_V PI10463_ and TcdB_R20291_, as well as antibodies newly developed by antibody phage display, revealed different characteristics of the yet poorly described delivery domain of TcdB harboring the second receptor binding region. In summary, we found that conserved Cys-2232 in TcdB indirectly contributes to toxin–receptor interaction.

## Introduction

The two large glucosyltransferases TcdA and TcdB from *Clostridioides difficile* (*C. difficile*) are the main pathogenicity factors leading to the clinical symptoms associated with *C. difficile* infections (CDI) (Just and Gerhard, 2004; Voth and Ballard, 2005). These toxins glucosylate cytosolic Rho GTPases which are master regulators of the actin cytoskeleton. Glucosylation-derived inhibition of Rho GTPases affects cell morphology of host cells as well as other dynamic, actin-mediated processes. The role of Rho GTPases in cell cycle, gene expression, regulation of the NADPH oxidase, cell polarity, and others dedicates TcdA- or TcdB-exposed cells to apoptosis, once the majority of these signaling proteins are glucosylated. Since the intestinal epithelium is the first line of target for TcdA and TcdB, these toxins induce loss of barrier function. This in turn triggers inflammatory processes, which clinically impose as diarrhea and, in worse case, pseudomembranous colitis or toxic megacolon.

To develop new therapeutics that directly aim at the toxins or that inhibit interaction of toxins with target cells, a detailed knowledge about functional toxin domains, toxin structure, or uptake mechanism into host cells is necessary. In recent years, progress has been made in elucidating the crystal structure of TcdA as well as of TcdB and also in identifying toxin receptors, especially for TcdB (LaFrance et al., 2015; Yuan et al., 2015; Tao et al., 2016). A paradigm has been solved by dissecting two separate receptor binding domains in TcdB (Genisyuerek et al., 2011; Olling et al., 2011), and it can be assumed that this is also the case for TcdA (Gerhard, 2016). Redundant receptors as well as different uptake routes explain why these toxins are so effective and no toxin-resistant cell has been described so far. What was originally described as AB-structure type (A means enzymatically active subunit, B means binding subunit) for TcdA and TcdB as well as for all other large clostridial glucosyltransferases has now evolved to an ABCD structure type. This term acknowledges the different features found in each toxin, such as the N-terminal glucosyltransferase activity (A), the C-terminal binding domain (B), the cutting domain (C) in charge of autoproteolytic release of the GTD, and the intermediate delivery domain (D) which includes a hydrophobic region for membrane insertion and also harbors a second and putative third receptor binding region (Aktories et al., 2017). Despite a lot of detailed knowledge about the structure of toxins and also of prerequisites on host cell side for uptake of toxins, very little is known about the dynamic of toxin binding to cell surfaces and conformational changes of toxins that are associated with binding and translocation.

We previously reported about an intramolecular association of N- and C-terminal domains of TcdA which is assumed to stabilize the toxin to protect it from extracellular premature cleavage (Olling et al., 2014). At least for TcdA we postulate different conformational requirements such as: (1) stable conformation in the intestinal luminal environment, (2) binding to first receptor, most probably to carbohydrate structures via CROP domain, (3) binding to a functional receptor to induce uptake, (4) pH-dependent conformational changes that (5) coordinate and allow autoproteolysis and membrane passing of at least the glucosyltransferase domain. Since this almost applies to all large clostridial glucosyltransferases, we looked out for highly conserved structural characteristics. Most interesting is the conserved cysteine 2236 in TcdA which can also be found in TcdB from clades I, III, IV, and V at position 2232 but not in TcdB from hypervirulent (clade II) strains. This is true for all sequenced clade II strains. Based on previous studies it is clear that cysteine 2232 is not essential for the function of TcdB, since deletion mutants of all toxins tested so far still induced cell rounding (Barroso et al., 1994; Genisyuerek et al., 2011; Olling et al., 2011). Here we evaluated cysteine 2232 in TcdB with respect to the conformation-associated functions, i.e., autoproteolysis, oligomerization, and receptor binding. To this end, we compared TcdB_V PI10463_ and TcdB_R20291_ and their complementary mutants TcdB_V PI10463_ C2232Y and TcdB_R20291_Y2232C.

## Materials and Methods

### Site Directed Mutagenesis of TcdA and TcdB Expression Constructs

Expression of recombinant proteins was done in *B. megaterium* expression system (MoBiTec). *Clostridioides difficile* TcdA_V PI10463_ was cloned into a modified pWH1520 vector (Burger et al., 2003), and TcdB_V PI10463_ and TcdB_R20291_ were cloned into pHis1522 vector (Wohlan et al., 2014). *C. difficile* strain R20291 for cloning of TcdB_R20291_ was obtained from the DSMZ (DSM-27147; NCTC 13366). Point mutation for exchange of amino acid residue 2232 in TcdB was performed via GeneTailor^TM^-PCR using Q5^®^ High Fidelity Polymerase (NEB) and mutagenic primers TcdB C2232Y and TcdB Y2232C according to the instruction manual of GeneTailor^TM^ Site-Directed Mutagenesis System (Invitrogen). Mutagenesis of Cys-2236 in TcdA_V PI10463_ was done via QuikChange II Site-Directed Mutagenesis Kit (Stratagene) according to the protocol provided by the supplier. Table 1 lists oligonucleotides used for mutagenesis. All constructs were sequenced for successful mutation. The plasmids pWH1520_TcdA_V PI10463_ C2236Y, pHIS1522_tcdB_V PI10463_ C2232Y, and pHIS1522_tcdB_R20291_ Y2232C were then transformed into *B. megaterium* WH320 protoplasts following the protocol provided by the supplier.

**Table 1:** Oligonucleotides used for mutagenesis.

Protocol	Primer	Base sequence (5′→ 3′)
GeneTailor	TcdB Y2232C_s	CCAGAAACTAAAAAAGCATGTAAAGGTATT AATGTAATTGATG
	TcdB Y2232C_a	ATGCTTTTTTAGTTTCTGGATCGAAATAA TATTTATC
	TcdB C2232Y_s	CCAGAAACTAAAAAAGCATACAAAGGTAT TAATTTAATTGATG
	TcdB C2232Y_a	ATGCTTTTTTAGGTTTCTGGATTGAAATAA TATTTATCAC
QuikChange	TcdA C2236G_s	GCTATTGCTGCAATTCATCTAGGCAC TATAAATAATGACAAG
	TcdA C2236G_a	CTTGTCATTATTTATAGTGCCTAGATGAAT TGCAGCAATAGC


### Purification of Recombinant Proteins

For each toxin one-liter cultures of the respective transformed *B. megaterium* culture was harvested, lysed by sonification, and centrifuged to isolate His-tagged toxins from the supernatant. Purification was done by gravity flow using Protino^®^ Ni-IDA Packed Columns (Macherey-Nagel). Afterward, elution buffer was exchanged with storage buffer (50 mM NaCl, 20 mM Tris–HCl, pH 8.0) using Zeba Desalting Spin Columns (Pierce). Purity and specific concentration of toxin were determined by SDS-PAGE.

### Cell Culture

HEp-2 cells were cultivated in Minimum Essential Medium Eagle medium (MEM) with 10% fetal bovine serum, 100 μM penicillin, and 100 μg/ml streptomycin. The culture was passaged twice a week after reaching 75% confluence. One day prior to experiments, the cells were seeded onto 24-well or 96-well microtiter plates to achieve 50% confluency for cell rounding and competition assays. Caco-2 cells were cultured in DMEM supplemented with 10% fetal bovine serum, 100 U/ml penicillin, and 100 μg/ml streptomycin. For the measurement of transepithelial electrical resistance (TEER), the cells were seeded onto filter inserts for cell culture and were allowed to grow and differentiate for 7–10 days. Only cell monolayer with an initial TEER of at least 100 Ω^∗^cm^2^ was used for experiments. The TEER was measured with an EVOM device (Millipore) equipped with an Endohm chamber (World Precision Instruments) for 12-well filter inserts.

### Cell Rounding and Competition Assay

For cell rounding assay, HEp2-cells were exposed to indicated toxins at given concentrations for 2 h at 37°C, which is the EC50 time for 100 pM TcdB. All toxin stock solutions were adjusted to the same specific toxin concentration according to SDS-PAGE prior to treatment of cells. Cell rounding was evaluated by microscopy, counting completely rounded cells per total cells in randomly selected areas at 20-fold magnification. To investigate the cytotoxic effect, higher concentrations of up to 10 nM were used. Therefore, the HEp2-cells were exposed to toxins for 4 h. Then, a DAPI solution [200 nM 4′,6-Diamidin-2-phenylindol in phosphate buffered saline (PBS)] was added to the culture medium for 15 min and DAPI-positive. The total number of cells were documented by fluorescence and phase contrast microscopy of the identical area, respectively. We repeated cytopathic and cytotoxic assays with these two mentioned concentrations three times (*n* = 8) under identical conditions. For neutralization assay, TcdB_V PI10463_ (300 ng in 1 ml culture medium) was incubated for 15 min at room temperature with 3 μg scFv-Fc. Afterward the complete toxin/antitoxin mixture was applied to HEp-2 cells in 24 wells and morphological changes were documented after 3 h incubation.

### Receptor Pull Down Experiments

Pull down assays were performed to show direct interaction of toxin with the known extracellular domains of the two known receptors FZD2 (aa glycine 24 – serine 156) and PVRL3. Both proteins were purchased as Fc-fusion proteins from ACROBiosystems. Fc-tagged receptors (1 μg) were bound to 10 μl Protein A/G sepharose beads. Beads loaded with receptors were blocked for 30 min with 1 mg/ml bovine serum albumin in PBS and subsequently washed two times with PBS. Immobilized receptor (1 μg) was incubated in 300 μl PBS containing 2 mg of the indicated toxin for 60 min on a rotator at 4°C. After washing the pellet beads with PBS three time, the beads were resolved in 30 μl Laemmli buffer, heated to 95°C, and subjected to SDS-PAGE along with samples from input and supernatant. Silver-stained gels were densitometrically evaluated, and the toxin precipitated in the beads fraction was calculated as percentage of input.

### SDS-PAGE and Silver Staining

Proteins were resolved in 7.5 or 10% polyacrylamide gels (SDS-PAGE) based on their molecular weight. Proteins in SDS gels were visualized by Coomassie staining. In case of autoproteolysis and pull-down assays we used the silver staining kit from ThermoFisher, Germany. Silver staining was done strictly according to the protocol supplied by the manufacturer.

### Immunoblot

For specific detection of proteins, we transferred the resolved proteins from SDS-PAGE onto nitrocellulose by semi-dry Western blot for 1 h at 17 V. In case of dot blot, indicated proteins (100 ng in 2 μl storage buffer) were directly spotted onto nitrocellulose. Free non-selective binding sites were blocked with 5% milk powder in Tris-buffered saline containing 0.2% Tween 20 (TBS-T) for 30 min. The appropriate first antibody was added to 0.5% milk powder in 1 × TBST. The nitrocellulose was incubated overnight at 4°C to allow antibody binding. After washing with TBS-T, the second antibody was added. The results were documented using Pierce^TM^ ECL Western Blotting Substrate SuperSignal West Femto from Thermo Scientific^TM^ and The Kodak Digital Science^TM^ Image Station 440CF (IS440CF) system.

### Inositol Hexakisphosphate-Induced Autoproteolysis

To start autoproteolysis assay, 1 μg of toxin was added to 100 μl assay buffer (1 mM dithiothreitol and 1 mM zinc chloride in PBS) and supplemented with indicated concentrations of D-myo-inositol 1,2,3,4,5,6-hexakisphosphate (InsP_6_). The samples were incubated for 1 h at 37°C, and the reaction was stopped by the addition of 5-fold Laemmli buffer and heating to 95°C for 5 min. Afterward, the samples were subjected to SDS-PAGE and subsequent silver staining to evaluate cleavage products.

### Generation of a Monoclonal Anti-TcdB Antibody

Antibodies against TcdB_V PI10463_ were selected in the scFv-format from the human naive antibody gene libraries HAL9/10 (Kugler et al., 2015). The selection and screening were performed as described earlier (Russo et al., 2018). In brief, for antibody selection, the scFv phage libraries HAL9/10 were incubated on a TcdB fragment (aa 1-1852) immobilized on Costar High-Binding microtiter plates (Sigma-Aldrich Chemie GmbH, Munich, Germany). For some pannings, a preincubation of the libraries on TcdB1-1128 was performed to select binders against the CROP domain. Three panning rounds were performed and 94 clones were screened on TcdB1–1852 by antigen ELISA using soluble scFvs. From the anti-TcdB scFv clones’ plasmid DNA was isolated, and the antibody DNA was sequenced. Subsequently, the unique scFv genes were re-cloned into pCSE2.6-hIgG1-Fc-XP using *Nco*I/*No*tI for mammalian production as scFv-Fc, an IgG-like antibody format. The production and purification were performed as described earlier (Jager et al., 2013).

### Statistics

All data analyses were performed with GraphPad Prism 5, version 5.02, (2008). Student’s *t*-test was applied for all analyses. *P*-values of <0.05 were considered as significant and indicated by asterisk (^∗^<0.05 and ^∗∗^<0.01). Mean values ± standard deviation are shown in all graphics.

## Results

### Cytopathic and Cytotoxic Potency of TcdB_V PI10463_ and TcdB_R20291_

**FIGURE 1:**
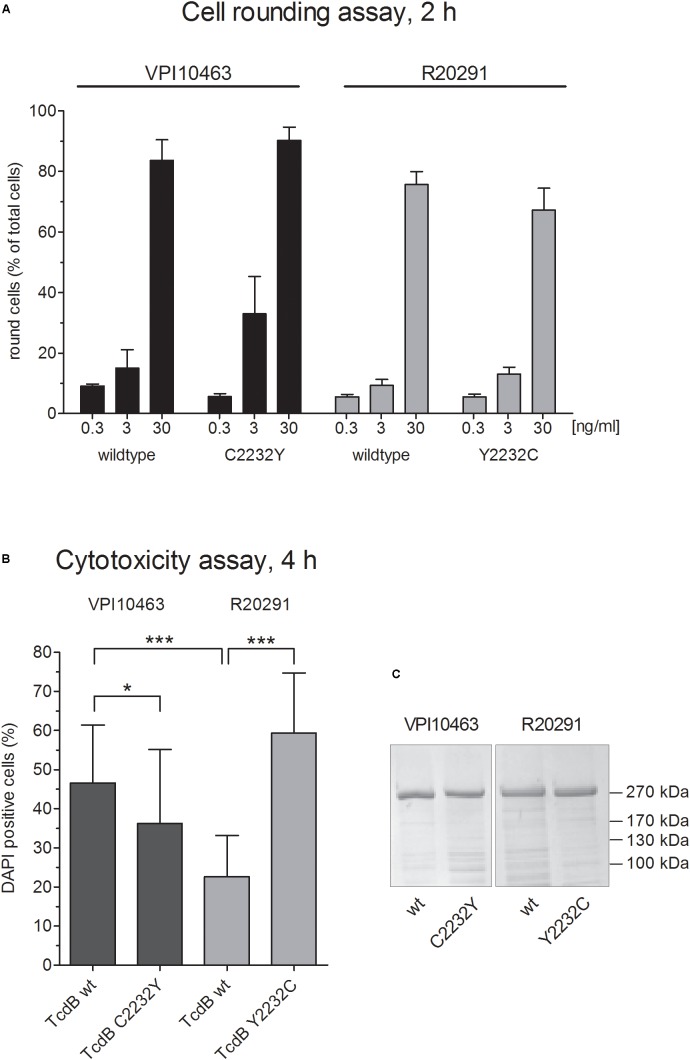
Cytopathic and cytotoxic effect of clade I and II TcdB. **(A)** Dose-dependent cytopathic effect of TcdB and TcdB C2232Y from strain VPI10463 (clade I) as well as TcdB and TcdB Y2232C from strain R20291 (Clade II). Shown are the percentage of rounded cells after treatment with TcdB at given concentrations for 2 h (means ± SD, *n* = 6). **(B)** Cytotoxic effect of indicated toxins after treatment of cells with 1 μg/ml for 4 h (means ± SD, *n* = 24). **(C)** Coomassie stained SDS-gel of all four toxins used in panels **(A,B)** showing comparable purity and concentration.

Based on negative stain data and crystal structures of TcdA and TcdB domains (Chumbler et al., 2016), as well as reports about intramolecular association of the C-terminal domain with the N-terminal part of TcdA (Olling et al., 2014; Zhang et al., 2015), we hypothesized that the conserved cysteine 2236 in TcdA and the homologous 2232 in TcdB contribute to the conformation of toxins. Cysteine 2232 is located in an exposed region of the CROP domain which might interact with the upstream located delivery domain. Interestingly, TcdB from hypervirulent *C. difficile* strains (clade II) possess a tyrosine instead of cysteine. The observation of different potencies of the reference TcdB and TcdB from hypervirulent strain prompted us to systematically investigate the role of the conserved cysteine 2232 in TcdB. Therefore, we generated inverse mutants of recombinant TcdB to exchange cysteine and tyrosine at position 2232 in both TcdB toxinotypes. First, we performed a cell rounding assay to compare the cytopathic effects followed by DAPI-incorporation assay to quantify the cytotoxic effect. We used HEp-2 cells, since these cells show transcriptome for all known TcdB receptors and are well described for early cell death induced by TcdB (Beer et al., 2018). We applied three different concentrations (0.3, 3, and 30 ng/ml resembling 1, 10, and 100 pM, respectively) and did not observe significant differences in wildtype toxins and their according mutant (Figure 1A). Thus, cysteine or tyrosine at position 2232 is not essential for biological function of reference TcdB from clade I (strain VPI10463) or clade II (hypervirulent strain R20291), respectively. From earlier studies we know that the intracellular flush of toxins is decisive for whether cytopathic or cytotoxic effect, i.e., early cell death, occurs (Beer et al., 2018). We therefore additionally tested the cytotoxic potency of all toxins (Figure 1B). Unlike cell rounding, induction of early cell death as measured by DAPI-incorporation was significantly altered. On the other hand, TcdB_V PI10463_ C2232Y showed weak but significantly decreased cytotoxic effect, and the Y2232C mutant of TcdB_R20291_ showed increased cytotoxic potency. We hypothesize that cysteine 2232 contributes to conformation of TcdB, thereby affecting receptor binding and uptake into cells. Comparison of wildtype TcdB from clade I and II, possessing either a cysteine or tyrosine at position 2232, respectively, supported this hypothesis. TcdB_R20291_ was significantly less cytotoxic compared to TcdB_V PI10463_ when applied to HEp-2 cells. Figure 1C shows comparable concentrations and purity of wildtype and mutant toxins.

### Exchange of Cys-2232 Has No Effect on Autoproteolysis

**FIGURE 2:**
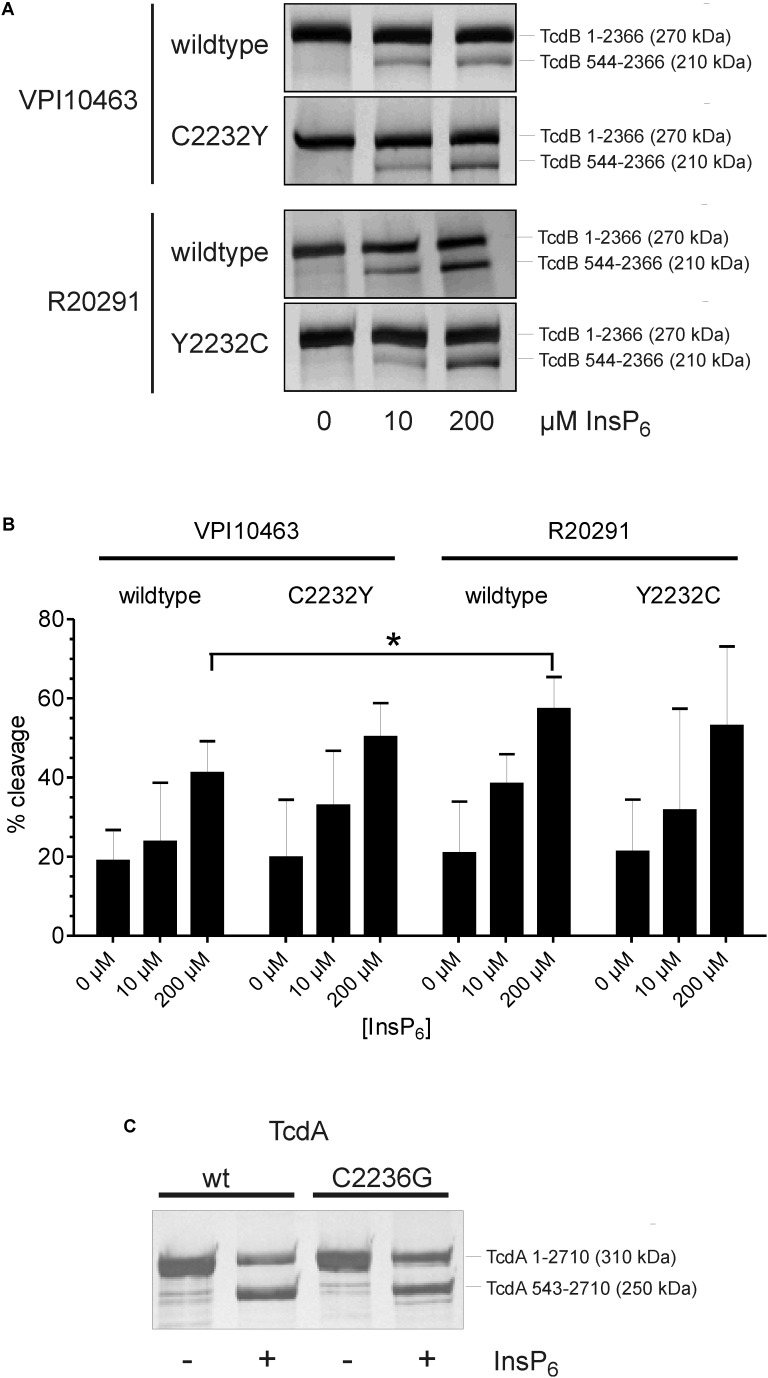
Autoproteolysis of TcdB. **(A)** Autoproteolytic cleavage of TcdB from strain VPI10463 and R20291 (2 μg each) is induced with different concentration of InsP_6_. Silver-stained gel shows full-length toxin (270 kDa) and the long cleavage product TcdB 544-2366, lacking the GTD (210 kDa). **(B)** Densitometrical evaluation of four separate cleavage experiments reveals no significant differences in cleavage of the mutated toxin compared with the wildtype form. Given are the percentage of cleaved product compared to full-length toxin (means ± SD, *n* = 4). **(C)** InsP6-induced cleavage of TcdA and TcdA C2236G (strain VPI10463). Silver-stained gel is representative for three independent experiments.

The C-terminal CROP domain in TcdA associates with the N-terminal part of the toxin (Olling et al., 2014; Zhang et al., 2015). Thereby, the CROP domain stabilizes the conformation in TcdA which provides protection from premature autoproteolytic cleavage. Extracellular cleavage results in inactivation of toxin due to loss of the GTD (Kreimeyer et al., 2011). It is reasonable that in TcdB, the CROPs stabilize the conformation in a similar manner to that in TcdA. To elucidate the impact of cysteine 2232 on conformation-dependent features, we performed *in vitro* cleavage experiments. In these experiments, wildtype TcdB_V PI10463_ and mutated TcdB_V PI10463_ C2232Y, as well as wildtype TcdB_R20291_ and mutated TcdB_R20291_ Y2232C, were compared (Figure 2). Full-length toxins as well as the cleaved long fragment lacking the GTD (aa 544-2366) were resolved in SDS-PAGE. Silver-stained proteins in gels were densitometrically evaluated (Figures 2A,B). InsP_6_ at low concentration of 10 μM induced weak cleavage of toxins (20–30%) after 1 h, which increased to about 50% in the presence of 200 μM InsP_6_. The autoproteolysis of TcdB_V PI10463_ or TcdB_R20291_ was not altered when amino acid residue at position 2322 was exchanged with tyrosine or cysteine, respectively. Obviously, this region does not significantly contribute to the overall conformation of toxin in a way that InsP_6_-induced autoproteolysis is affected. Nevertheless, our data were in line with a previous report, showing that processing of TcdB from hypervirulent strain is significantly more effective than of historical/reference TcdB (Lanis et al., 2012). Subsequent autoproteolysis assay with TcdA and TcdA C2236G validated the results found for TcdB (Figure 2C). Cleavage assay for TcdA requires different InsP_6_ concentration since this toxin is more resistant to autoproteolysis (Kreimeyer et al., 2011; Olling et al., 2014). Here we chose InsP_6_ concentrations that allow detection of increase as well as decrease in autoproteolysis. Similar to TcdB, the presence or absence of the conserved cysteine does not affect autoproteolysis in *in vitro* assay.

### Amino Acid Residue 2232 Contributes Indirectly to Receptor Binding

**FIGURE 3:**
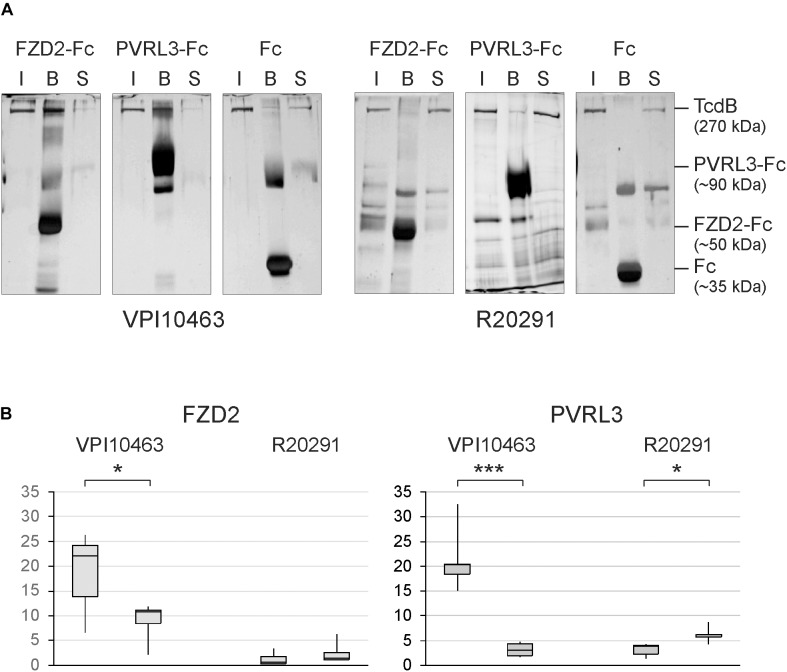
Receptor binding of clade I and II TcdB. **(A)** Silver-stained SDS gels show binding of TcdB to the immobilized extracellular domains of FZD2 and PVRL3. Shown is the input (I), the fraction bound to Fc-fusion receptors coupled to protein A/G sepharose beads (B) and toxin remaining in the supernatant (S) from pull-down assays. Controls were performed with only Fc coupled to protein A/G sepharose. **(B)** Densitometrical evaluation of binding of TcdB and TcdB C2232Y from strain VPI10463 as well as TcdB and TcdB Y2232C from strain R20291 to FZD2-Fc and PVRL3-Fc from pull-down assays (means ± SD, *n* = 6, ^∗^,^∗∗∗^ *p*-values < 0.05 and < 0.001, respectively.

Based on the findings, that mutated TcdB alters the cytotoxic potency of TcdB, we investigated the molecular interaction of TcdB with two of the known receptors, FZD2 and PVRL3. The receptor binding region in TcdB for both receptors is upstream of the CROP domain (aa 1830-2366) (Orth et al., 2014; Manse and Baldwin, 2015; Tao et al., 2016; Chen et al., 2018). Thus, the amino acid sequence around point mutation at position 2232 does not directly interact with these receptors. The silver-stained gels showed detectable binding of TcdB_V PI10463_ to immobilized Fc-fusion proteins of FZD2 extracellular domain, as well as to PVRL3. A complementary decrease of toxin was observed in the supernatant after pull down of beads (Figure 3A). Unspecific precipitation of toxins was probed with Fc-loaded beads. Surprisingly, TcdB_R20291_ showed only weak binding to PVRL3 and no binding to FZD2. TcdB_R20291_ differs from TcdB_V PI10463_ mainly in three clusters between aa 1770 and 1811 and also in phenylalanine 1597 (serine 1597 in TcdB_R20291_) which is part of the FZD2 binding region (Chen et al., 2018). Although the FZD2 and PVRL3 receptor binding region in TcdB is upstream of the CROP domain and cysteine 2232 or tyrosine 2232 are supposedly not directly involved in receptor interaction, we found that point mutation at this position affects binding to FZD2 and PVRL3. Binding to both the receptors was reduced in TcdB_V PI10463_ when cysteine 2232 was changed to tyrosine (Figure 3B). Accordingly, when tyrosine 2232 was exchanged for cysteine in TcdB_R20291_, at least in PVRL3 binding, a small but significant increase was detected. Obviously, the CROP domain contributes to the exposing/covering of the non-CROP receptor binding region.

### Competition Assays

**FIGURE 4:**
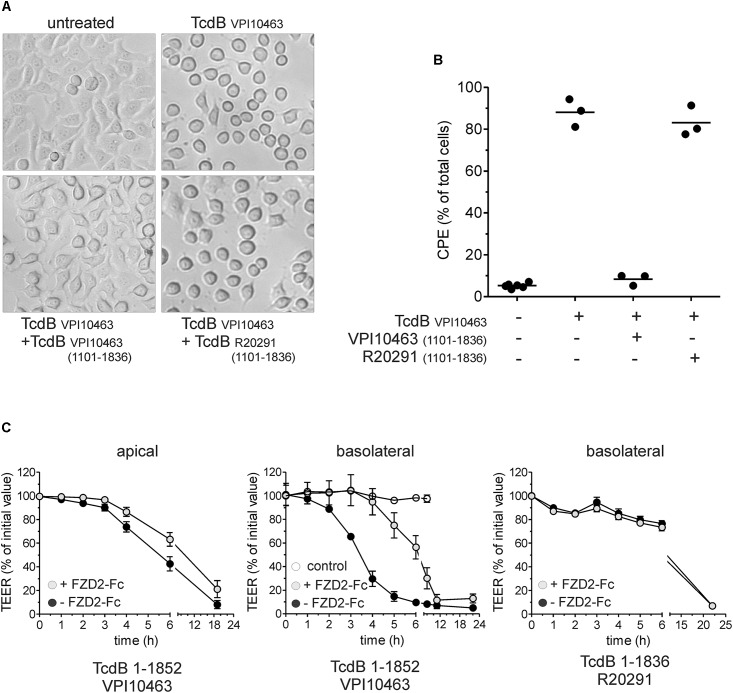
Competition assay with receptor binding domain and frizzled 2 extracellular domain. **(A)** Micrographs show HEp-2 cells treated with 300 ng/ml TcdB_V PI10463_ for 3 h. Competition was performed by treatment of cells with TcdB in the presence of 1,000-fold molar excess of the receptor binding fragment TcdB_V PI10463_ 1101–1836 or TcdB_R20291_ 1101–1836. **(B)** Quantitative evaluation of micrographs from three independent experiments. Shown is the percentage of rounded cells (cytopathic effect; CPE). **(C)** Effect of CROP-truncated TcdB on transepithelial electrical resistance (TEER) as surrogate for barrier function. TcdB_V PI10463_ 1–1852 (100 pM, black circles) reduces TEER to about 50% within 6 h when apically applied. Addition of FZD2-Fc in a 1000:1 ratio slightly delayed TcdB effect on TEER (gray circles). TcdB_V PI10463_ 1–1852 (30 pM, black circles) reduces TEER to about 50% within 3.5 h when applied from basal (middle graph). Addition of FZD2-Fc in a 1000:1 ratio delayed TcdB effect for 3 h TEER (gray circles; middle graph). 30 nM TcdB_R20291_ 1–1836 reduced TEER for only 25% within 6 h, and was not affected by addition of FZD2-Fc (1000:1 ratio; right graph) (mean ± SD, *n* = 3).

The amino acid sequence 1750–1850 in TcdB is of importance when comparing clade I and II TcdB (Hunt and Ballard, 2013; Larabee et al., 2017a,b). Since this region is involved in receptor binding, we performed competition assays to elucidate the functional differences between both toxins. First, we pre-incubated cells for 3 min with the full delivery domain of TcdB_V PI10463_ (aa 1101–1836), which includes receptor binding regions for FZD2 as well as for PVRL3 but not for CSPG4. TcdB_V PI10463_ 1101–1836, but not TcdB_R20291_ 1101–1836, reduced the cytopathic effect induced by 300 ng/ml TcdB_V PI10463_ in HEp-2 cells when given in 1,000-fold molecular excess (Figure 4A). After 3 h, 90% of the cells showed complete rounding when treated with TcdB_V PI10463_ alone, whereas roughly 10% rounded up in the presence of TcdB_V PI10463_ fragment 1101–1836. Even 1,000-fold excess of TcdB_R20291_ 1101–1836 could not reduce cell rounding induced by TcdB_V PI10463_ in more than 80% of the cells according to quantitative evaluation of micrographs (Figure 4B). In a complementary approach, we applied the extracellular domain of FZD2 fused to the Fc-domain to intercept cell surface binding. This competition assay was performed with Caco-2 cells lacking CSPG4, an important receptor which interacts with the region around the beginning of the CROP domain (Yuan et al., 2015; Gupta et al., 2017). Due to the lack of CSPG4, the Caco-2 cells allow TcdB entry only via FZD1,2,7, and PVRL3. Caco-2 cells were grown on filter inserts to measure the TEER as read out system for TcdB-induced morphological changes (Figure 4C). For this assay, we used the CROP-depleted toxin fragments TcdB_V PI10463_ 1-1852 and TcdB_R20291_ 1-1836. We found that TcdB_V PI10463_ 1-1852 is less potent when applied from the apical side. 20 μg/ml FZD2-Fc slightly delayed the weak TcdB-induced decrease in TEER when applied apically. When TcdB_V PI10463_ 1–1852 was applied from the basolateral side, a 50% decrease in TEER was achieved after 3.5 h of incubation, which was delayed for 3 h in the presence of FZD2-Fc. This result indicates asymmetrical expression of FZD2 on intestinal epithelial cells, constituting a receptor for targeting cells mainly from the basolateral side. Application of TcdB_R20291_ 1–1836 revealed that this toxin is less potent on Caco-2 cells from basolateral than reference TcdB_V PI10463_. Furthermore, the extracellular domain of FZD2 had no detectable effect on the weak decrease in TEER induced by TcdB_R20291_ 1–1836. These results clearly indicate substantial differences in non-CROP receptor binding of clade I and II TcdB. This result is in line with the different interaction of TcdB from clade I and II in receptor pull-down assays.

### Conformation-Dependent Detection and Neutralization of Toxins by scFv-Fc

**FIGURE 5:**
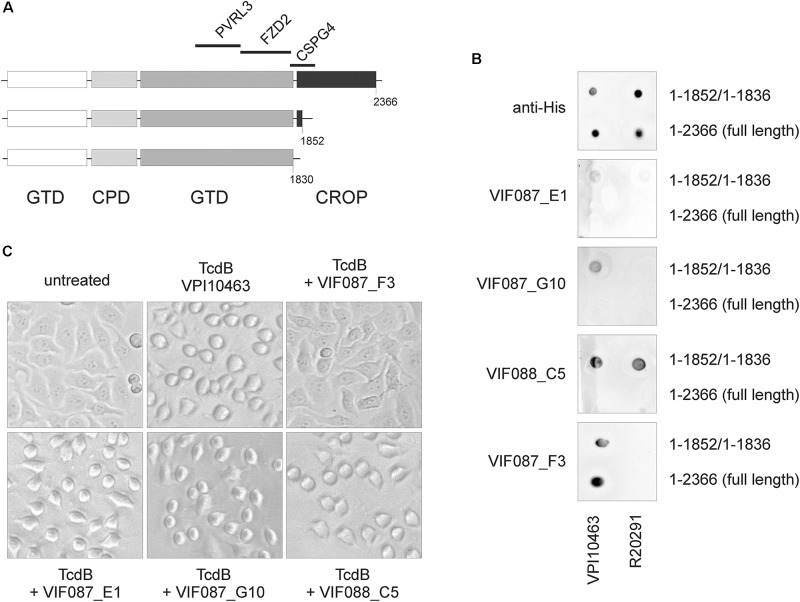
Detection and neutralization of TcdB by specific scFv-Fc. **(A)** Scheme of TcdB showing deletion mutants and specific receptor binding regions. TcdB 1-1852 does not bind CSPG4, although partial CSPG4 binding region is left. **(B)** Dot blot analysis of full-length toxins (TcdB_V PI10463_ 1–2366; TcdB_R20291_ 1–2366) and CROP-truncated toxins (TcdB_V PI10463_ 1–1852; TcdB_R20291_ 1–1836) using various scFv-Fc as well as anti-His antibody as positive control under non-denaturing conditions. Full-length TcdB was only detected by ViF87_F3. ViF88_C5 was the only scFv-Fc that recognized epitope in both clade I and clade II toxins, albeit only in CROP-depleted toxins. Shown are the representative results of 2–3 independent experiments. **(C)** All four scFv-Fc were applied in neutralization assays. Only VIF087_F3 prohibited cell rounding induced by 300 ng/ml TcdB_V PI10463_ in HEp-2 cells for 3 h.

Since the region up-stream of the CROP domain is essential for receptor binding, we addressed the question whether antibodies directed against this part of toxin might have neutralizing effects. Several scFv-Fc were generated by antibody phage display as reported by Fühner and coworkers in the same issue of this journal, four of which were tested here in dot blot and cell rounding assay (cross reference). First, TcdB_V PI10463_ and TcdB_V PI10463_ 1–1852, as well as TcdB_R20291_ and TcdB_R20291_ 1–1836, were spotted onto nitrocellulose and detected by anti-His antibody (Figure 5A). All toxins were recognized via C-terminal His-tag to similar extent. The antibodies ViF087_E1 and ViF087_G10 showed only weak binding to TcdB_V PI10463_ 1-1852 but not to full-length TcdB_V PI10463_. TcdB from hypervirulent strain R20291 was not recognized. Neither full-length nor CROP-truncated toxin was bound by both antibodies. Only ViF088_C5 recognized an epitope in both TcdB toxinotypes, albeit only when the CROP domains were deleted. ViF087_F3 is the only antibody that recognizes TcdB in the presence and absence of the CROP domain. ViF087_F3, however, is specific for TcdB_V PI10463_ and does not recognize TcdB_R20291_. All four antibodies generated by phage display were additionally tested in neutralization assay for their capacity to prevent uptake of TcdB_V PI10463_ into HEp-2 cells (Figure 5B). TcdB_V PI10463_ (300 ng/ml) induced almost complete cell rounding in cells after 3 h. ViF087_F3 scFv-Fc, which was the only scFv-Fc from selection that tested positive for recognizing full-length TcdB, showed a neutralizing effect. Cells treated with the combination of TcdB_V PI10463_ and ViF087_F3 scFv-Fc showed complete unaffected control morphology after 3 h of incubation. None of the other scFv-Fc antibodies inhibited TcdB-induced cell rounding. Together, these data indicate that specific epitope regions within the N-terminal toxin fraction (amino acid region 1–1850) are not necessarily accessible for antibodies in native TcdB (and therefore not for cell surface receptors) due to tertiary conformation.

## Discussion

The present study is about the conformation of TcdB, which was indirectly assayed by different functional read outs. Here, we wanted to learn about the role of the prominent cysteine 2232 which is conserved in TcdA and TcdB, but is exchanged for tyrosine in TcdB from hypervirulent strains. We generated the corresponding mutant toxins TcdB_V pi10463_ C2232Y and TcdB_R20291_ Y2232C and found that mutated toxins were as potent as their wildtype forms when tested in cell rounding assay. Interestingly, we did not observe that TcdB_R20291_ from hypervirulent strain exhibited higher potency than reference TcdB_V pi10463_ in Hep-2 cells as could be expected (Lanis et al., 2010, 2012). Moreover, TcdB_R20291_ was even significantly weaker in cytotoxicity regarding induction of early cell death (Ecd) than TcdB_V pi10463_. Most astonishing was that the replacement of cysteine 2232 by tyrosine reduced cytotoxicity in TcdB_V pi10463_ and complementary to this, exchange of tyrosine 2232 in TcdB_R20291_ with cysteine increased cytotoxicity. This is the first evidence that a single amino acid within the Crop domain systematically affects a specific function of TcdB. The mechanism by which TcdB induces Ecd is not known. Knockdown of the TcdB receptor Pvrl3/nectin-3 prohibits Ecd (LaFrance et al., 2015). According to Manse and Baldwin (2015) the receptor binding region for Pvrl3 in TcdB is between amino acids 1250 and 1804, indicating that the cysteine 2232 has to contribute indirectly to receptor binding. Very recently we reported about the uptake efficiency as prerequisite for Ecd rather than a specific receptor-mediated effect (Beer et al., 2018), which is not in contradiction with Pvrl3 knockdown result. Even from this point of view our present results can be interpreted the same: The Crop domain modulates non-Crop domain receptor binding, thereby modulating uptake of toxin into cells.

To be sure that no other conformation-dependent feature account for different cytotoxicities, we performed an autoproteolysis assay, but found that mutated TcdB showed the same InsP6-dependent cleavage efficiency as the corresponding wildtype form. Moreover, clade II TcdB showed enhanced cleavage efficiency in our study but less cytotoxic effect in terms of ECD. Thus, autoproteolytic release of the GTD is most likely not the reason for different cytotoxic effects, which was also reported by Chumbler et al. (2012).

The most novel finding is that the interaction of TcdB_R20291_ with FZD2 extracellular domain and with PVRL3 is strongly reduced compared to historical TcdB_V PI10463_. Both receptors bind TcdB upstream of the CROPs, as shown by CROP-truncated TcdB (Manse and Baldwin, 2015; Tao et al., 2016). Very recently the exact binding region of the FZD2 extracellular cysteine-rich domain (FZD2-CRD) was defined in TcdB (Chen et al., 2018). This is close to the region where cluster of mutations are found in TcdB_R20291_, and it can be assumed that the differences in primary amino acid sequence contribute to lack of interaction of FZD2-CRD with this toxinotype. Especially phenylalanine 1597 directly interacts with FZD2 {Chen}, which is replaced by serine in TcdB_R20291_. The interaction of PVRL3 with TcdB_R20291_ was comparable to TcdB_V PI10463_ C2232Y. In line with this, TcdB_R20291_ Y2232C showed increased binding to PVRL3, although not to an extent as wildtype TcdB_V PI10463_. Our results show that the receptor binding regions upstream of the CROPs are affected by the CROP domain. We assume that the CROP domain affects accessibility of the receptor binding region for FZD2 and PVRL3 and that cysteine 2232 contributes to the arrangement of the CROPs. As mentioned in the Introduction, receptor interaction and receptor binding region in TcdB are targets for therapeutic intervention to treat CDI symptoms. To date, only Actoxumab and Bezlotoxumab are the antibodies used for trial therapy in CDI with different efficiencies (Wilcox et al., 2017). Only Bezlotoxumab is approved for therapy, since Actoxumab did not pass the clinical trial due to efficacy and safety reasons (Merck, 2015). Both antibodies recognize epitopes within the CROP domain (Orth et al., 2014; Hernandez et al., 2017). Here we tested antibodies (scFv-Fc) recombinantly generated by antibody phage display for their binding to TcdB. To get antibodies directed against the delivery domain, we used CROP-deleted TcdB 1–1852 for selection with (ViF087) or without (ViF088) pre-absorbance of antibodies to TcdB 1–1128 fragment. Our approach resulted in antibodies that only bind to TcdB immobilized onto a nitrocellulose membrane in the absence of the CROP domain, implicating that the CROP shields antigenic epitopes within the N-terminal portion (aa 1–1852) of TcdB. The only exception was ViF87_F3, which also recognizes full length TcdB. This was the only scFv-Fc which also showed neutralizing capacity in a cell rounding assay. Obviously, the translocation domain (aa 1101–1852) is not accessible for specific antibodies. Especially the region of amino acids 1750–1850, which harbors important cluster specific for clade I or clade II TcdB, contributes to toxin conformation and intrinsic peptides can inactivate TcdB (Larabee et al., 2017a). Additionally, the interaction of this hypervariable region as shown by Larabee et al., 2015 impacts protein–protein interaction within TcdB and the exposure of neutralizing epitopes. Our results from dot blot are not in line with the results from antigen ELISA as reported by HUST/Fühner in the same issue of this journal (here: insert cross reference). This can be explained by the binding of toxin to different matrices or partial denaturation which might lead to exposure of a further epitope. Our studies extend this model by including the C-terminal CROP-domain which might contribute not only to intramolecular interaction but also to intermolecular interaction.

In summary, we conclude that clade II toxins, e.g., TcdB_R20291_, interact much lesser with FZD2 and also with PVRL3 than TcdB_V PI10463_. This finding implicates significant differences in susceptibility of different cells toward clade I and clade II toxins. The three-dimensional conformation of toxin is affected when the conserved cysteine 2232 within the CROP domain is exchanged, indirectly influencing non-CROP receptor binding epitopes. Further studies have to investigate if intermolecular association or oligomerization contributes to receptor selection.

## Author Contributions

S-YC and DS carried out all the experimental work and performed data analysis. VF and MH generated scFv-Fc. HT and L-AB provided toxins. RG designed and supervised the study. S-YC and RG wrote the initial manuscript. All the authors contributed to the final manuscript.

## Conflict of Interest Statement

The authors declare that the research was conducted in the absence of any commercial or financial relationships that could be construed as a potential conflict of interest.
